# Apigenin enhances skeletal muscle hypertrophy and myoblast differentiation by regulating Prmt7

**DOI:** 10.18632/oncotarget.20962

**Published:** 2017-09-16

**Authors:** Young Jin Jang, Hyo Jeong Son, Yong Min Choi, Jiyun Ahn, Chang Hwa Jung, Tae Youl Ha

**Affiliations:** ^1^ Division of Nutrition and Metabolism Research, Korea Food Research Institute, Seongnam, Republic of Korea; ^2^ Division of Food Biotechnology, University of Science and Technology, Daejeon, Republic of Korea

**Keywords:** apigenin, Prmt7, GPR56, skeletal muscle hypertrophy, myoblast differentiation, Gerotarget

## Abstract

Apigenin, a natural flavone abundant in various plant-derived foods including parsley and celery, has been shown to prevent inflammation and inflammatory diseases. However, the effect of apigenin on skeletal muscle hypertrophy and myogenic differentiation has not previously been elucidated. Here, we investigated the effects of apigenin on quadricep muscle weight and running distance using C57BL/6 mice on an accelerating treadmill. Apigenin stimulated mRNA expression of MHC (myosin heavy chain) 1, MHC2A, and MHC2B in the quadricep muscles of these animals. GPR56 (G protein-coupled receptor 56) and its ligand collagen III were upregulated by apigenin supplementation, together with enhanced PGC-1α, PGC-1α1, PGC-1α4, IGF1, and IGF2 expression. Prmt7 protein expression increased in conjunction with Akt and mTORC1 activation. Apigenin treatment also upregulated FNDC5 (fibronectin type III domain containing 5) mRNA expression and serum irisin levels. Furthermore, apigenin stimulated C2C12 myogenic differentiation and upregulated total MHC, MHC2A, and MHC2B expression. These events were attributable to an increase in Prmt7-p38-myoD expression and Akt and S6K1 phosphorylation. We also observed that Prmt7 regulates both PGC-1α1 and PGC-1α4 expression, resulting in a subsequent increase in GPR56 expression and mTORC1 activation. Taken together, these findings suggest that apigenin supplementation can promote skeletal muscle hypertrophy and myogenic differentiation by regulating the Prmt7-PGC-1α-GPR56 pathway, as well as the Prmt7-p38-myoD pathway, which may contribute toward the prevention of skeletal muscle weakness.

## INTRODUCTION

Muscle mass and strength are reduced with aging, movement decrease, and obesity. [1, 2]. Muscle loss is closely associated with acute and chronic disease, increased insulin resistance, and rheumatoid arthritis [3]. Moreover, skeletal muscle strength is inversely associated with all-cause mortality in men [4]. Prevention of muscle loss may therefore contribute to improved quality of life, and attenuate chronic disease and mortality for the aged.

Muscle loss is associated with the decreased regenerative capacity of skeletal muscle stem cells, although the exact molecular mechanism responsible for the loss of myofibers remains to be elucidated [5]. MyoD (Myoblast determination protein) initiates differentiation to the skeletal muscle lineage and the formation of myoblasts [6]. The activity of MyoD is regulated by promyogenic signaling pathways which include p38 mitogen-activated protein kinase (p38 MAPK) and Akt [5, 7, 8]. Skeletal muscle hypertrophy refers to the increase in muscle mass that occurs following the enlargement of pre-existing skeletal muscle fibers [9]. Both IGF1 and IGF2 regulate skeletal muscle development and adult muscle regeneration and hypertrophy [10], while Akt and mTORC1, downstream effectors of IGF1, are critical regulators of skeletal muscle hypertrophy [11]. The modulation of muscle stem cell differentiation and skeletal muscle hypertrophy represent potential strategies for the prevention of muscle loss.

Prmt7 (protein arginine methyltransferase 7) is highly expressed in skeletal muscle, and regulates muscle oxidative metabolism. Prmt7 modulates PGC-1α expression *via* interaction with and activation of p38MAPK [12]. Prmt7 is also required for the maintenance of muscle stem cell regenerative capacity [13]. PGC-1α4 has been found to be highly expressed in exercising skeletal muscle, which increases IGF1 expression, and induces skeletal muscle hypertrophy [14]. The G protein-coupled receptor 56 (GPR56) is a transcriptional target of PGC-1α4 and is induced by resistance exercise [15]. Overexpression of GPR56 increases IGF-1 expression *via* Gα12/13 and activates mTORC1, which results in muscle hypertrophy [15].

Apigenin (4’,5,7-trihydroxyflavone) is a natural flavone abundant in various edible plants including parsley, celery, chamomile, oranges, and grapefruit [16]. The anti-inflammatory effects of apigenin have been investigated for its potential use as a therapy to prevent inflammation-related disease [17]. Apigenin also suppresses prostate cancer progression, inhibits cancer cell proliferation, and induces apoptosis in various cancer cells [18-20]. The neuroprotective and memory improvement effects of apigenin have been demonstrated in animal studies [21, 22]. Recently, apigenin was also reported to attenuate dyslipidemia, hepatic steatosis, and insulin resistance in high-fat diet induced obese mice [23]. It was previously shown that apigenin prevents LPS-induced atrogin-1 expression in C2C12 myotubes by regulating phosphorylation of JNK [24]. However, the effect of apigenin on skeletal muscle hypertrophy and myogenic differentiation remains to be elucidated.

In the present study, we investigated the effect of apigenin on skeletal muscle hypertrophy and its molecular mechanisms of action in C57BL/6 mice. The C2C12 myoblast line is a well-established model for the study of myogenic differentiation [25]. We also sought to elucidate the molecular mechanisms responsible for the effect of apigenin on myogenic differentiation in C2C12 cells.

## RESULTS

### Connectivity mapping identifies apigenin as a candidate small molecule for the prevention of muscle loss

To investigate functional relationships between drug candidates and diseases, a connectivity map resource has been created [26, 27]. Previous studies using connectivity maps have identified ursolic acid and tomatidine as small molecule inhibitors of skeletal muscle atrophy [28, 29]. To identify small molecules for the potential prevention of muscle loss, we used a skeletal muscle mRNA profile of an elderly male (70-80 years old) and compared it to that of a young male (19-25 years old) [30]. An mRNA profile of the aged muscle and mRNA expression patterns in the presence of 1309 small molecules using several human cancer cell lines were compared using the connectivity map, and we discovered 870 small molecules showing reverse similarity. Affymetrix GeneChip Human Genome HF-U133A array data are available in the ArrayExpress database (www.ebi.ac.uk/arrayexpress) under accession number E-GEOD-1428. Among the small molecules showing a negative correlation with the mRNA profile of aged muscle, we selected several flavonoids (apigenin, hesperetin, kaempferol, luteolin, myricetin, quercetin) and investigated whether they increase mRNA expression of the myosin heavy chain (MHC). We found that only apigenin significantly increased mRNA expression of MHC2A (Supplementary Figure S2). The connectivity score of apigenin was -0.465 in the PC3 cell line. The workflow used to identify apigenin is summarized in Supplementary Figure S1.

### Apigenin increases quadricep muscle weight and improves muscle function

**Figure 1:**
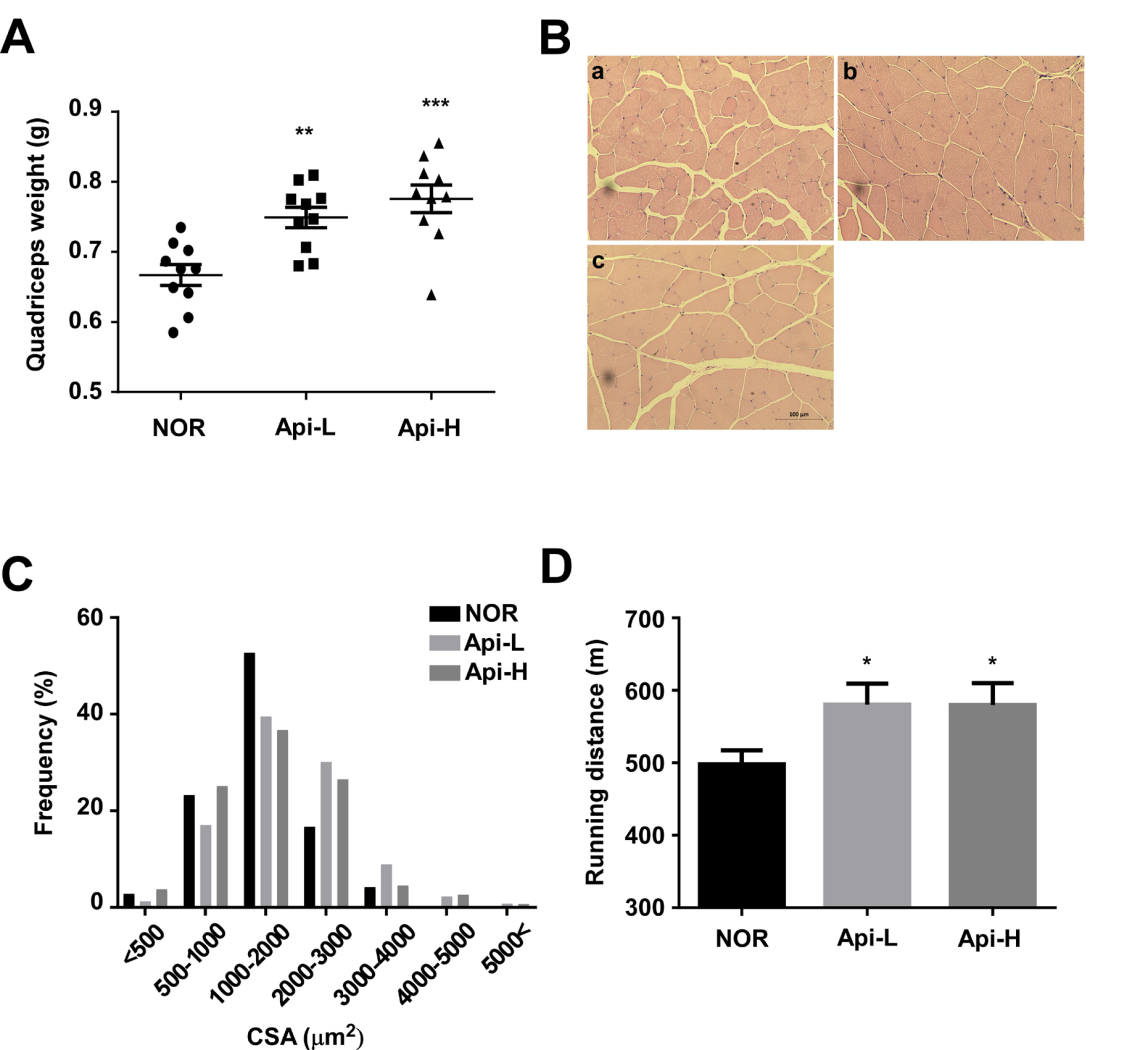
Effect of apigenin on quadricep muscle weight and treadmill running distance **A.**-**D.** Male C57BL/6 mice were provided *ad libitum* access to standard diet or standard diet supplemented with 0.2% apigenin (Api-L) or 0.4% apigenin (Api-H) for 7 weeks. **A.** Apigenin increased quadricep muscle weight in a dose-dependent manner. Each point represents one animal, and horizontal bars indicate mean ± SEM. **B.**-**C.** Apigenin promoted quadricep muscle fiber hypertrophy. **B.** Representative images of H&E staining of sections of quadricep muscle; a:NOR, b:Api-L, c:Api-H **C.** Frequency distribution of muscle fiber cross-sectional area (CSA) **D.** Apigenin enhanced running distance on the accelerating treadmills. NOR: normal group, Api-L: apigenin 0.2% diet group, Api-H: apigenin 0.4% diet group. Data represent means ± SEM. * *p <* 0.05, ** *p <* 0.01, ***p < 0.001 *versus* the normal group.

**Figure 2:**
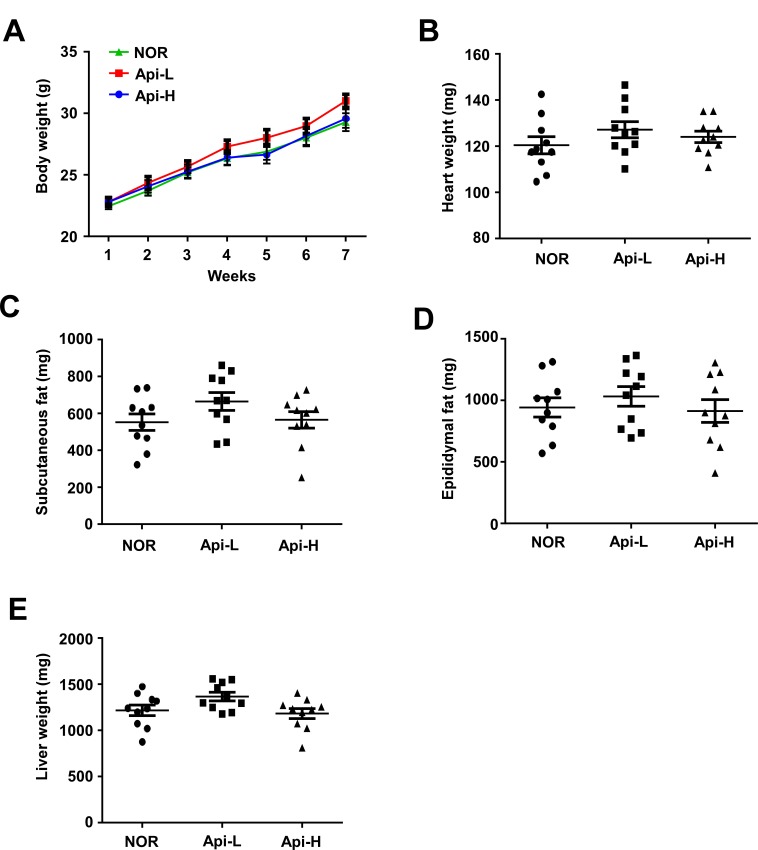
Effect of apigenin on weights of total body, heart, subcutaneous fat, epididymal fat, and liver Male **A.**-**E.** C57BL/6 mice were provided *ad libitum* access to standard diet or standard diet supplemented with 0.2% apigenin (Api-L) or 0.4% apigenin (Api-H) for 7 weeks. The weights of total body, heart, subcutaneous fat, epididymal fat, liver were not altered by apigenin supplementation. Each point represents one animal, and horizontal bars represent mean ± SEM.

To further investigate the effect of apigenin on skeletal muscle hypertrophy, mice were permitted *ad libitum* access to either a standard diet or a standard diet containing 0.2% apigenin (Api-L) or 0.4% apigenin (Api-H) for 7 weeks. In the mice receiving apigenin, quadricep muscle weight increased in a dose-dependent manner (Figure 1A). Results of hematoxylin and eosin (H&E) stains of quadricep cross-sections confirmed that apigenin increased muscle fiber size (Figure 1B-1C). The weight of the tibialis anterior, gastrocnemius, and triceps muscle were not altered by apigenin (Supplementary Figure S3). Dietary apigenin also increased running distance on an accelerating treadmill (Figure 1D). However, apigenin did not significantly affect total body weight, heart, subcutaneous fat, epididymal fat, or liver weight (Figure 2A-2E).

### Apigenin upregulates mRNA expression of MHC and GPR56 signaling in mouse quadricep muscle and increases serum irisin levels

**Figure 3:**
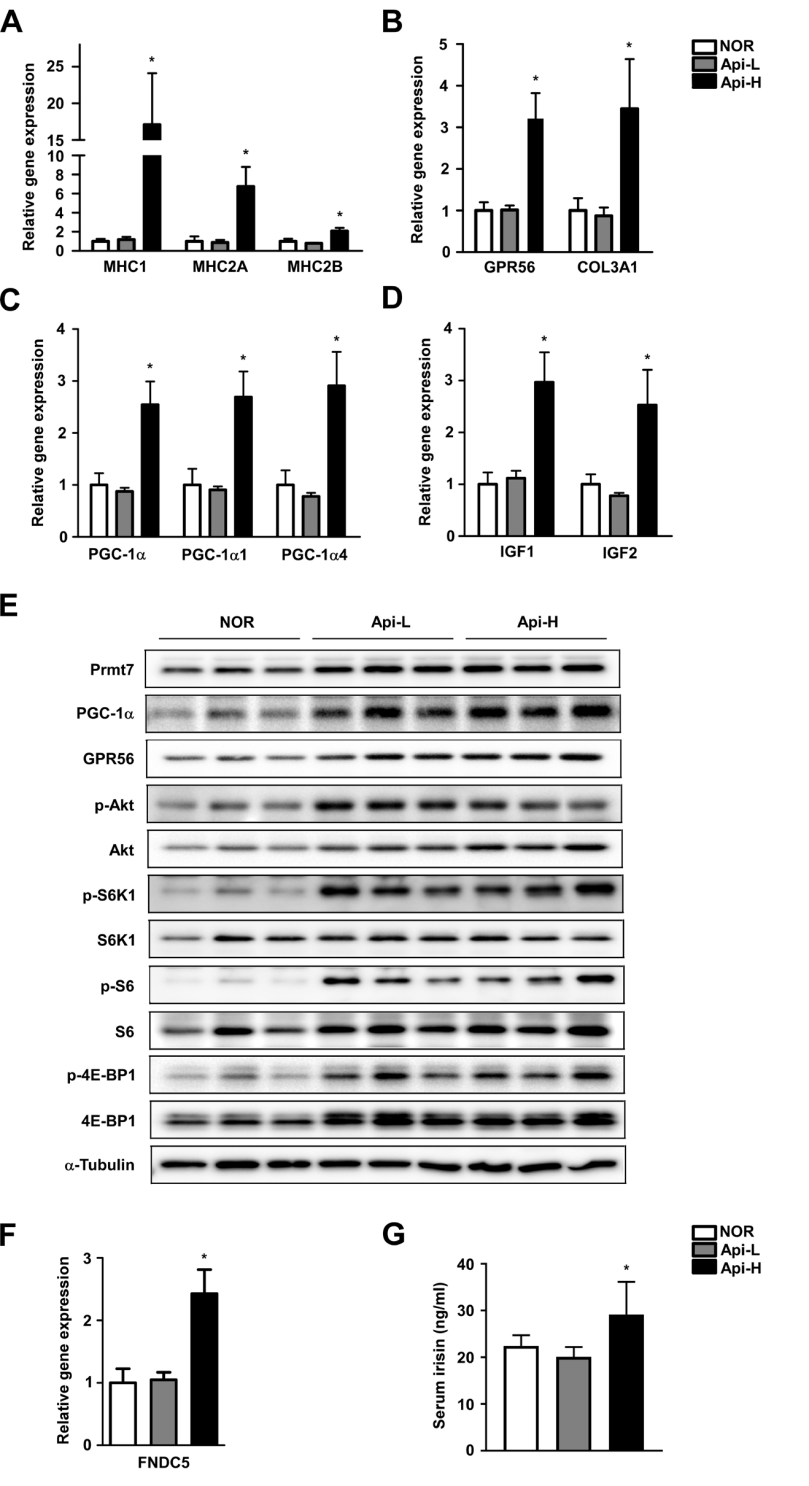
Effect of apigenin on muscle fiber type, GPR56 pathway signaling, and irisin concentration **A.**-**G.** Male **A.**-**D.** C57BL/6 mice were provided *ad libitum* access to standard diet or standard diet supplemented with 0.2% apigenin (Api-L) or 0.4% apigenin (Api-H) for 7 weeks. Skeletal quadricep muscles were harvested for further analysis. **A.** Apigenin promotes mRNA expression of MHC1, MHC2A, and MHC2B. **B.** Apigenin increases GPR56 and Col3a1 mRNA expression. **C.** Apigenin stimulates total PGC-1a, PGC-1a1, and PGC1a-4 mRNA expression. **D.** Apigenin increases mRNA expression of IGF1 and IGF2. mRNA expression was quantified by qPCR. **E.** Apigenin enhances protein expression of Prmt7, PGC-1α, and GPR56, and activation of mTORC1. Protein expression was analyzed by Western blot. **F.**-**G.** Apigenin increases FNDC5 mRNA expression and irisin concentration in mouse serum. Serum irisin levels were quantified by ELISA assay. Data are means ± SEM. * *p <* 0.05 *versus* the normal group.

To investigate whether apigenin alters muscle fiber type, mRNA expression of MHC1, 2a and 2b were measured. We found that mRNA expression of MHC1, 2A, and 2B were significantly increased in the Api-H group (Figure 3A). GPR56 is a major regulator of mechanical overload-induced muscle hypertrophy [15]. Apigenin increased mRNA and protein expression of GPR56 and mRNA expression of its ligand, collagen III (Figure 3B and 3E). Apigenin also enhanced mRNA and protein expression of total PGC-1α, and mRNA expression of PGC-1α isoforms 1 and 4 (Figure 3C). IGF1 is downstream of PGC1a4-GPR56 and promotes myotube hypertrophy [15]. We observed that mRNA expression of IGF1 and 2 were increased by apigenin supplementation (Figure 3D). FNDC5 (fibronectin type III domain-containing protein 5) is a precursor of irisin, and the Api-H group exhibited increased FNDC5 expression and serum irisin concentrations (Figure 3F and 3G).

### Apigenin increases Prmt7 expression and activates the mTORC1 pathway in mouse quadricep muscle

Prmt7 is a critical regulator of muscle mass in aging, and controls PGC-1α activity [12]. We observed that apigenin supplementation increases Prmt7 protein expression (Figure 3E). mTORC1 plays a key role in skeletal muscle anabolism and protein synthesis [31]. Akt, a downstream effector of IGF and an upstream regulator of mTORC1, was observed to be phosphorylated in the presence of apigenin. Apigenin increased phosphorylation of S6K1 and 4E-BP1, two major substrates of mTORC1. Phospho-S6, a direct substrate of S6K1, was also increased by apigenin in mouse quadricep muscle (Figure 3E).

### Apigenin promotes myogenic differentiation

**Figure 4:**
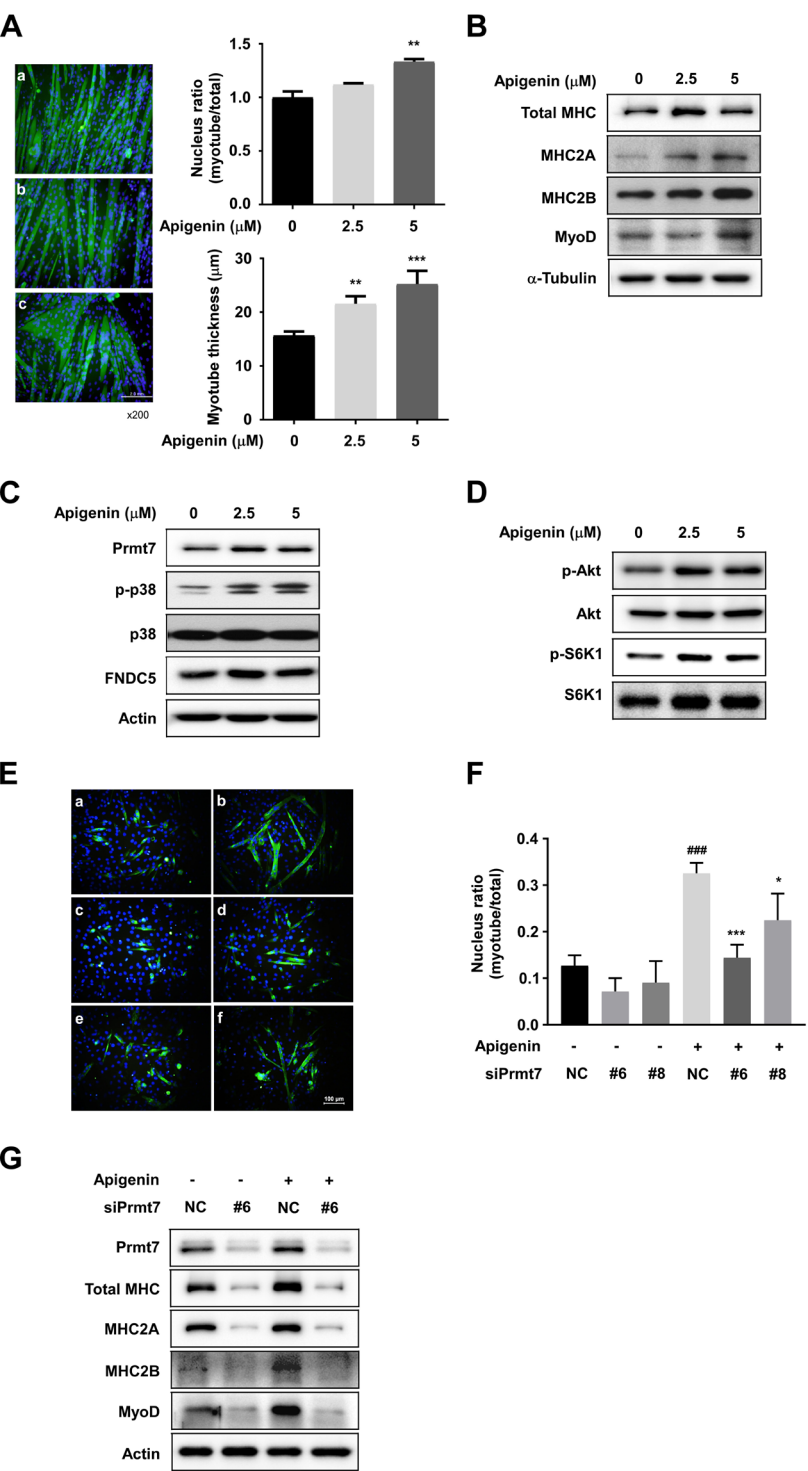
Effect of apigenin on myogenic differentiation **A.** Apigenin stimulates myogenic differentiation. a: cont, b: apigenin 2.5µM, c: apigenin 5 µM. C2C12 cells were differentiated in the presence or absence of apigenin for 6 days. After 6 days of differentiation, cells were fixed and stained with total MHC antibody. Nucleus ratio was calculated as the average number of nuclei in MHC positive multinucleated cells above total nuclei. Myotube thickness was calculated as the average diameter of MHC-positive multinucleated myotubes. ** *p <* 0.01, *** *p <* 0.001 *versus* control. **B.** Apigenin increases total MHC, MHC2A, MHC2B and myoD expression. **C.** Apigenin promotes Prmt7, phospho-p38, FNDC5 expression. **D.** Phosphorylation of Akt and S6K1 are stimulated by apigenin treatment. C2C12 cells were differentiated in the presence or absence of apigenin for 2 days. Protein expression was analyzed by Western blot. **E.** Apigenin enhances myogenic differentiation through regulating Prmt7. a: negative control (NC), b: control plus apigenin, c: siPrmt7 #6, d: siPrmt7 #8 e: siPrmt7 #6 plus apigenin, f: siPrmt7 #8 plus apigenin. **F.** Nuclear ratio was calculated as the average number of nuclei in MHC positive multinucleated cells above total nuclei. ^###^ *p <* 0.001 *versus* NC, * *p <* 0.05, *** *p <* 0.001 *versus* NC plus apigenin. **G.** Apigenin upregulated MHC, MHC2A, MHC2B, and myoD expression through Prmt7. C2C12 cells were transfected with siPrmt7 or NC, and differentiated in the presence or absence of apigenin for 2 days.

To examine the effect of apigenin on myogenic differentiation, C2C12 myoblasts were differentiated into myotubes in the presence or absence of apigenin, and stained with total MHC antibody. The nuclear ratio is defined as the number of MHC-positive multinucleated cells divided by the total number of nuclei in a cell population. Apigenin at 5µM increased the nucleus ratio in C2C12 myotubes, and myotube thickness was enhanced in a dose-dependent manner by apigenin treatment (Figure 4A). At the early differentiation stage, 2.5 µM of apigenin enhanced myogenic differentiation similar to the effect of 10ng/ml of IGF1 (Supplementary Figure S4). Western blot analysis showed that apigenin increased protein expression of total MHC, MHC2A, and 2B (Figure 4B). MyoD is an important factor to determine the differentiation potential of an activated myoblast to drive differentiation [32]. We found that MyoD protein expression was stimulated by apigenin treatment (Figure 4B). These results suggest that apigenin stimulates myogenic differentiation.

### Apigenin modulates Prmt7, p38MAPK and the Akt signaling pathway

To identify the molecular mechanisms responsible for apigenin’s effects on myogenic differentiation, we examined the effect of apigenin on p38 MAPK. We observed that apigenin increased phosphorylation of p38 MAPK (Figure 4C). A previous study has shown that knockdown of Prmt7 reduces MHC and myogenin expression, as well as p38 phosphorylation [12]. We also found that apigenin enhances protein expression of Prmt7 (Figure 4C), and increased the phosphorylation of Akt and S6K1, substrates of mTORC1 (Figure 4D).

### Prmt7 regulates the GPR56-mTORC1 pathway

**Figure 5:**
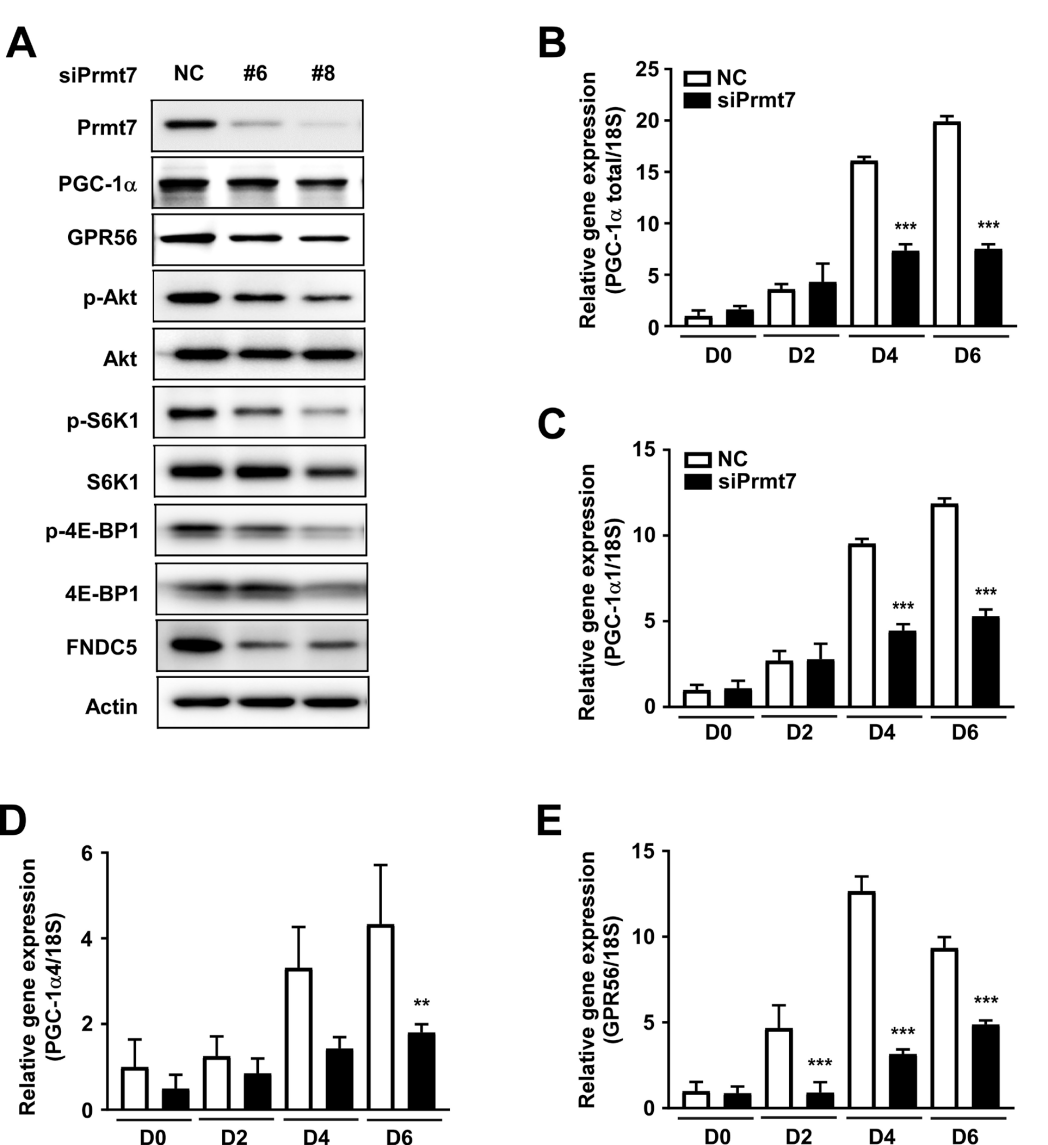
Prmt7 regulates the GPR56 pathway **A.** Knockdown of Prmt7 decreases GPR56 expression, mTORC1 pathway signaling, and FNDC5 expression. C2C12 cells were transfected with two different siPrmt7 constructs or a negative control (NC). After 48 hours, protein expression was analyzed by Western blot. **B.**-**E.** Prmt7 regulates PGC-1α and GPR56 mRNA expression. C2C12 cells were transfected with siPrmt7 or NC and differentiated for 2, 4, or 6 days. mRNA expression was quantified by qPCR. Data are expressed as means ± S.D. ** *p <* 0.01, *** *p <* 0.001 *versus* NC.

To investigate the correlation between Prmt7 and the GPR56 pathway, C2C12 cells were transfected with siPrmt7, and the expression of GPR56 and activation of its downstream signaling molecules were measured. Four different siRNAs were tested for knockdown effectiveness, and two siRNAs were selected. We confirmed that Prmt7 protein expression was decreased by two different individual siPrmt7 sequences. PGC-1α expression was attenuated by Prmt7 knockdown, consistent with a previous study [12]. GPR56 expression and phosphorylation of Akt, S6K1, and 4E-BP1 were attenuated by Prmt7 knockdown (Figure 5A). FNDC5 expression was also repressed by Prmt7 knockdown (Figure 5A). To determine whether Prmt7 regulates the expression of PGC-1α isoforms or GPR56, C2C12 myoblasts were transfected with siRNAs against Prmt7. The results showed that the knockdown of Prmt7 attenuates total PGC-1α, PGC-1α1, PGC-1α4, and GPR56 mRNA expression (Figure 5B-5E).

## DISCUSSION

Several studies have shown that various phytochemicals can stimulate skeletal muscle hypertrophy and myogenic differentiation. For example, resveratrol was reported to induce myogenesis and hypertrophy in murine myoblasts [9]. Similarly, phytoecdysteroid is found in spinach and a variety of herbs such as *Cyanotis vaga* and stimulates skeletal muscle hypertrophy *via* estrogen receptor beta [33]. Ursolic acid is found in apples and induces skeletal muscle hypertrophy and growth by enhancing IGF-1 signaling [28]. Tetrahydropalmatine and dehydrocorydaline were also reported to promote myogenic differentiation *via* p38MAPK [5, 34]. Tomatidine stimulates skeletal muscle hypertrophy and anabolism by activating the mTORC1 pathway [29]. These findings suggest that supplementation with phytochemicals in addition to specific proteins or amino acids may represent a potential therapeutic strategy for the prevention of muscle loss.

Previous studies have investigated the effects of several flavones including naringenin, hesperetin, epicatechin, apigenin, luteolin, kaempferol, daidzein, genistein, and delphinidin on LPS-induced atrogin-1 expression, a muscle-specific ubiquitin-ligase required for muscle atrophy [24, 35]. Among these nine flavones, apigenin showed the most potent atrogin-1 inhibition effect from 10 µM. Apigenin inhibits aromatase, an enzyme that converts androgens, including testosterone, into estrogen and is used to treat hormone-receptor positive breast cancer patients [36, 37]. However, 20-25% of women treated with aromatase inhibitors experience side effects such as joint pain, muscle weakness, and fragility [38]. In the present study, we found that apigenin increases skeletal muscle hypertrophy and myogenic differentiation. Apigenin may therefore be useful in the treatment of estrogen sensitive breast cancer to prevent muscle loss and weakness.

Muscle fibers are categorized into two major types, slow twitch type 1 muscle fibers and fast twitch type 2 muscle fibers. Type 1 is an oxidative muscle fiber that contains MHC1, and type 2 is a fast oxidative/glycolytic fiber that contains MHC2A and the fast-glycolytic fiber MHC2B [39]. Type 1 fibers are more susceptible to inactivity and denervation-induced atrophy, while type 2 fibers are more susceptible to malignancy, diabetes, chronic heart failure, and aging [39]. We observed that apigenin promotes increased expression of MHC1, MHC2A and MHC2B, implying that apigenin may have the potential to prevent inactivity-induced atrophy and age-related sarcopenia.

PGC-1α isoforms play several different roles in the regulation of skeletal muscle [14]. PGC-1α1 is induced by endurance exercise and regulates skeletal muscle oxidation, and PGC-1α4 is induced by resistance exercise and modulates skeletal muscle hypertrophy and strength. Prmt7 is known to upregulate PGC-1α expression, however, it is not known how Prmt7 modulates the PGC-1α isoforms. We found in the present study that Prmt7 regulates both PGC-1α1 and PGC-1α4. Irisin is released by muscle cells and has been reported to induce PGC-1α4 mRNA levels *via* autocrine action [40]. Upregulation of PGC-1α4 increases both GPR56 ligand secretion and GPR56 expression [15]. Subsequently, the increase in GPR56 expression stimulates IGF expression at the transcriptional level [15]. However, there has been no evidence of a direct link between Prmt7 and FNDC5. Our results show that knockdown of Prmt7 significantly reduces FNDC5 expression, whereas knockdown of FNDC5 does not appear to affect the expression of Prmt7. Moreover, knockdown of FNDC5 did not affect PGC-1α, PGC-1α4, or GPR56 expression, or phospho-Akt levels, although phopho-S6K1 and 4E-BP1 were attenuated (Supplementary Figure S5). These results suggest that FNDC5 or irisin are minor effects on PGC-1α4 regulation of Prmt7. That is, other pathway independent FNDC5 is more critical for Prmt7 to modulate PGC-1α4, although Prmt7 regulates FNDC5.

**Figure 6:**
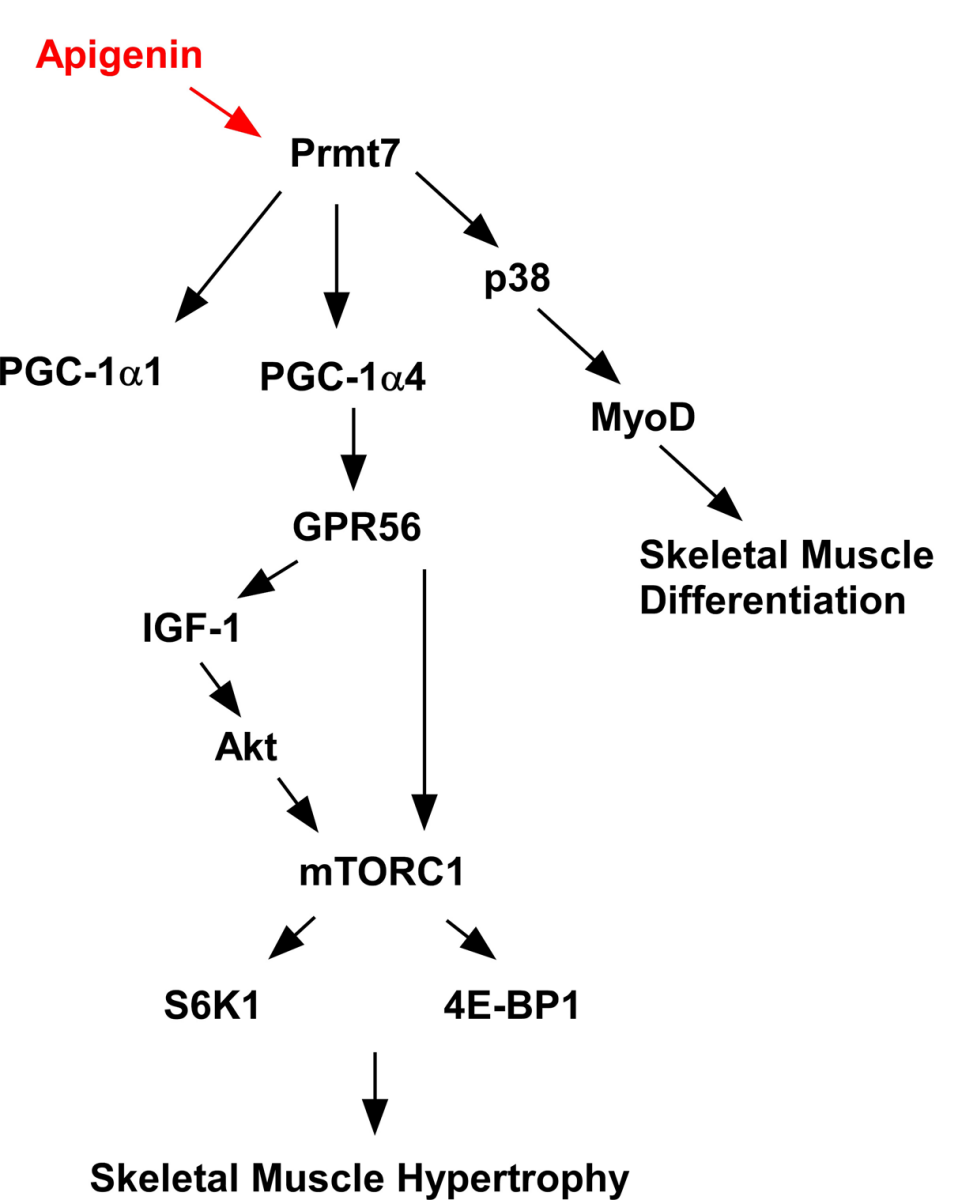
Proposed mechanism of action for the effects of apigenin on skeletal muscle

We found that apigenin increases quadricep muscle weight in mice and increases their running distance on an accelerating treadmill. These events are attributable to upregulation of the Prmt7-PGC-1α-GPR56 signaling pathway. We also found that apigenin induces myogenic differentiation by regulating Prmt7-p38-myoD and the Akt-S6K1 pathway in C2C12 cells. The proposed mechanism by which apigenin modulates skeletal muscle hypertrophy and myogenic differentiation is summarized in Figure 6. These results suggest that apigenin could be used as a functional food to prevent muscle loss and enhance muscle function. Further studies on the effect of apigenin in aged mouse muscle are needed to better understand the protective effect of apigenin on sarcopenia.

## MATERIALS AND METHODS

### Chemicals

Apigenin was purchased from Sigma Chemical (St Louis, MO, USA). Antibodies against total myosin heavy chain (MHC, MF20), MHC2a (SC-71), and MHC2b (BF-F3) were purchased from the Developmental Studies Hybridoma Bank, University of Iowa (Iowa City, IA, USA). Antibodies against Prmt7 (#14762), phospho-p38 (#9211), p38 (#9212), phospho-Akt (ser473) (#4056), Akt (#9272), phospho-S6K1 (Thr389) (#9234), S6K1 (#2708), pS6 (Ser235/236) (#2211), S6 (#2317), p4E-BP1 (Thr37/46) (#2855), and 4E-BP1 (#9644) were obtained from Cell Signaling Biotechnology (Beverly, MA, USA). Anti-MyoD (sc-760), GPR56 (sc-99089), GAPDH (sc-25778), and α-tubulin (sc-5386) were purchased from Santa Cruz Biotechnology (Dallas, TX, USA). PGC-1α (ab106814) and FNDC5 (ab174833) antibodies were obtained from Abcam (Cambridge, MA, USA).

### Animal model

All animal experiments were performed according to procedures approved by the Institutional Animal Care and Use Committee of the Korea Food Research Institute (KFRI-IACUC, #2016-0011). Male C57BL/6 mice (5 weeks old, *n* = 30) were maintained at a constant temperature (22 ± 2 °C) and kept on a 12-h light/12-h dark cycle with free access to food and water. After 1 week of adaptation, the animals were divided into three groups; normal, apigenin low, and apigenin high. The experimental diets were based on modified American Institute of Nutrition (AIN)-76A diets (Dyets, Bethlehem, PA, USA) with 30% sucrose. Apigenin low and high groups were fed the modified AIN-76A diet with 0.2% and 0.4% apigenin, respectively, for 7 weeks. Body weights were measured weekly.

### H&E staining

Mice quadricep muscles were fixed in 10% neutral-buffered formalin, embedded in paraffin, and 5 µm sections were prepared. Quadricep sections were stained with H&E. Images were captured with Olympus BX51 and cross-section area was quantified with IMT iSolution DT 9.2 software.

### Analysis of running distance on treadmill

Prior to exercise, mice were acclimated to a motor-driven open treadmill with a shock grid (Daejong Instrument Industry) for 20 min per day for 2 days. During acclimation, the treadmill speed was set at 10m/min and the treadmill incline was set at 15%. On the third day, exercise tolerance was tested: the shock grid was set at 50V and the treadmill incline was set at 15%. For the first 20 min of testing, the treadmill speed was set at 10 m/min. Every 2 min thereafter, the treadmill speed was increased by 2m/min. Running was terminated when mice contacted the shock grid for a duration exceeding10s.

### Cell culture, differentiation, and siRNA transfection

C2C12 myoblast cells were purchased from the ATCC (American Type Culture Collection, Manassas, VA. USA) and cultured in Dulbecco’s modified Eagle’s medium (DMEM) containing 10% fetal bovine serum (FBS), 100 U/ml penicillin and 100 μg/ml streptomycin (Invitrogen, Carlsbad, CA, USA) at 37°C under 5% CO_2_. Confluent cells were exposed to differentiation medium (DMEM with 2% horse serum) in the presence of either 2.5 or 5 μM of apigenin. The medium was replaced every 2 or 4 days. For knockdown of Prmt7 and FNDC5, C2C12 cells were transfected using RNAiMAX with two different siRNAs against Prmt7 or FNDC5 according to the manufacturer’s protocol.

### Immunofluorescence microscopy

C2C12 cells were differentiated in 12-well plates, fixed with 4% formaldehyde for 15 min, permeabilized with 0.1% Triton X-100 in phosphate-buffered saline (PBS), and blocked with 3% bovine serum albumin in PBS. The cells were then stained with total MHC antibody, followed by an Alexa Fluor 488-conjugated secondary antibody (Cell Signaling Biotechnology), and DAPI (4’,6-diamidino-2-phenylindole). Images were captured and processed with Olympus IX71 and Olympus DP controller 3.1.1 software.

### RNA extraction and real-time PCR

Total RNA extraction was performed using NucleoSpin RNAII(Macherey-Nagel, Düren, Germany), and cDNA was synthesized using ReverTra Ace qPCR RT Master Mix (Toyobo, Osaka, Japan). Quantitative real-time PCR was performed with the SYBR Green Master Mix (Toyobo, Osaka, Japan). The primer sequences are listed in Supplementary Table S1.

### Quantification of irisin in mice serum

Irisin in mouse serum was measured using commercially-available ELISAs (Phoenix Pharmaceuticals, Burlingame, CA, USA).

### Western blot assay

Western blot analysis was performed as previously described [41]. Briefly, the cells were lysed with RIPA buffer (Thermo Fisher Scientific, Rockford, IL, USA) and sonicated for 15 seconds at 40 W. Equal amounts of protein were subjected to 10% sodium dodecyl sulfate-polyacrylamide gel electrophoresis and transferred to polyvinylidenedifluoride (PVDF) membranes (Millipore, Billerica, MA, USA). The membranes were incubated with primary antibody followed by an HRP-conjugated secondary antibody (Santa Cruz Biotechnology, Santa Cruz, CA, USA). The protein bands were visualized using a chemiluminescence reagent (Amersham Pharmacia Biotech, Piscataway, NJ, USA).

### Statistical analysis

Data were analyzed with GraphPad Prism 6.0 version (GraphPad Software, Inc, La Jolla, CA). Data are expressed as means ± SEM for *in vivo* assays and ± SD. for *in vitro* assays. One-way ANOVA was used for statistical analyses followed by Dunnett’s multiple comparison test. A probability value of *p* < 0.05 was used as the criterion for statistical significance.

## SUPPLEMENTARY MATERIALS FIGURES AND TABLE


