# Supplementary material for: Apigenin enhances skeletal muscle hypertrophy and myoblast differentiation by regulating Prmt7
Source: Oncotarget. 2017 Sep 16;8(45):78300–11. doi: 10.18632/oncotarget.20962 (PMC5667963; doi:10.18632/oncotarget.20962)
Supplement: Supplementary file 1 [file oncotarget-08-78300-s001.pdf]

Apigenin enhances skeletal muscle hypertrophy and myoblast differentiation by regulating Prmt7

SUPPLEMENTARY MATERIALS

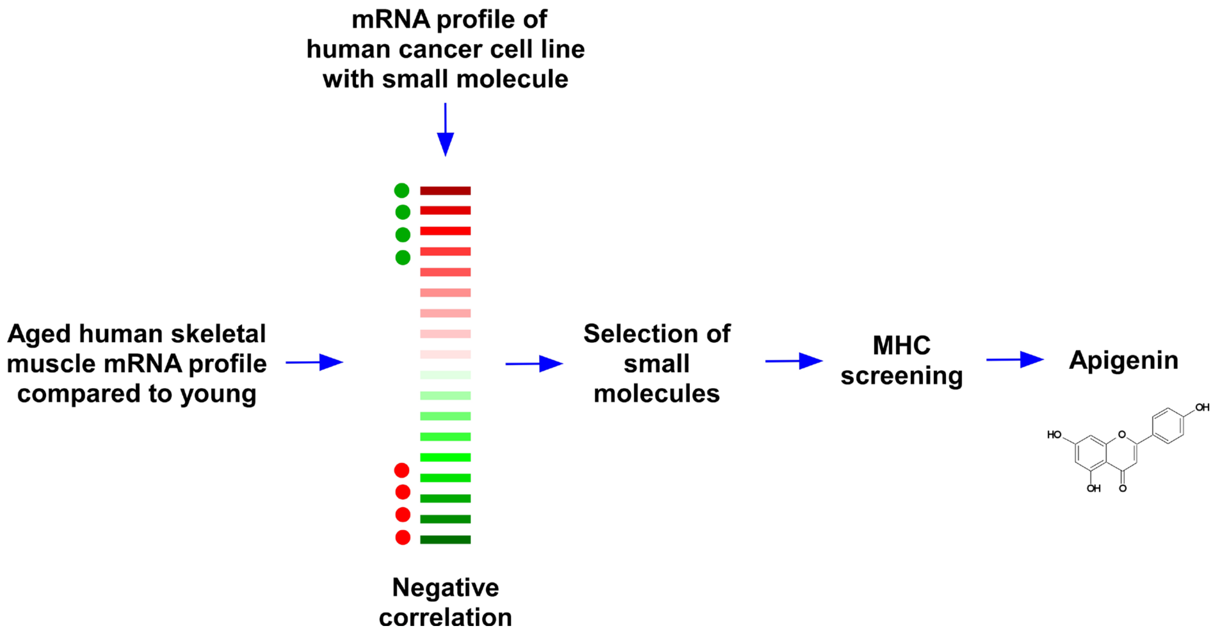

Supplementary Figure 1: Workflow used to identify apigenin

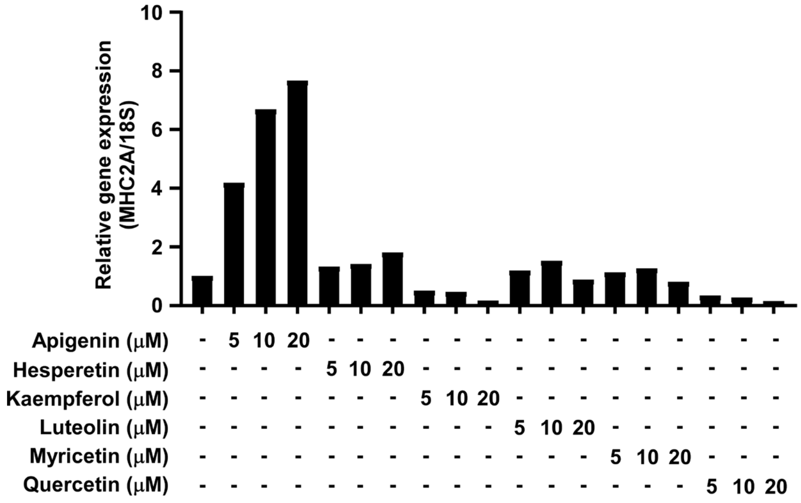

Supplementary Figure 2: Effects of various flavonoids on MHC2A expression. C2C12 cells were differentiated in the presence or absence of six flavonoids for 6 days. mRNA expression was quantified by qPCR.

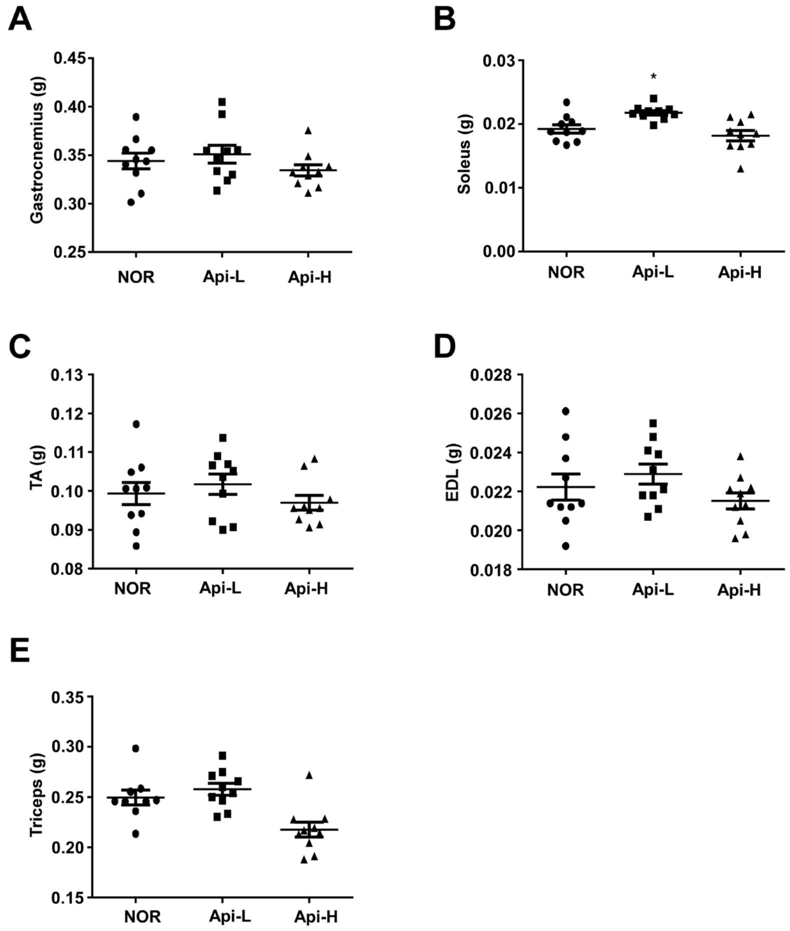

Supplementary Figure 3: Effect of apigenin on weights of gastrocnemius, soleus, tibialis anterior (TA), extensor digitorum longus (EDL), triceps. C57BL/6 mice were provided *ad libitum* access to standard diet or standard diet supplemented with 0.2% apigenin (Api-L) or 0.4% apigenin (Api-H) for 7 weeks. Each point represents one animal, and horizontal bars represent mean ± SEM. * $p < 0.05$ versus the normal group.

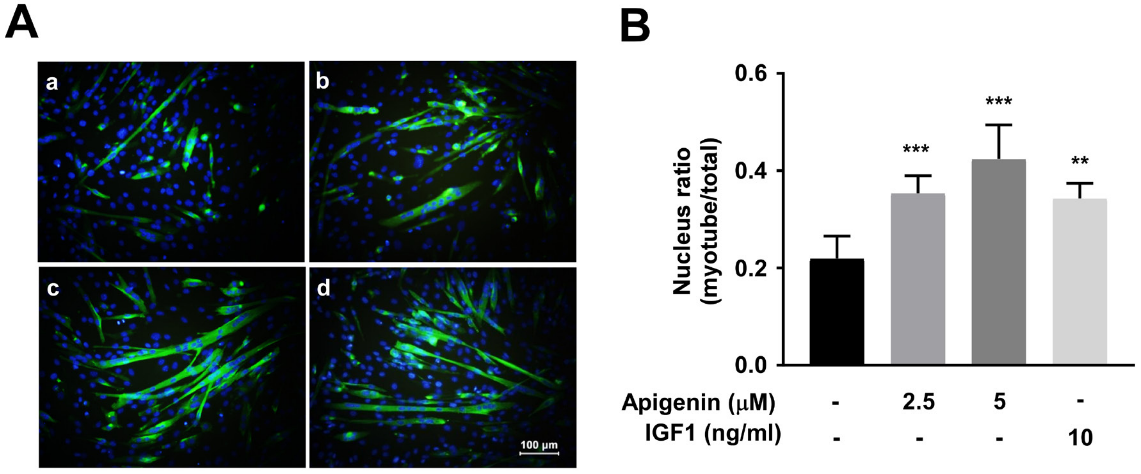

Supplementary Figure 4: Effect of apigenin compared to IGF1 on myogenic differentiation. (**A**) Apigenin and IGF1 stimulate myogenic differentiation. a: cont, b: apigenin 2.5μM, c: apigenin 5μM, d: IGF 10ng/ml. C2C12 cells were differentiated in the presence or absence of apigenin or IGF1 for 2 days. After 2 days of differentiation, cells were fixed and stained with total MHC antibody. (**B**) Nuclear ratio was calculated as the average number of nuclei in MHC positive multinucleated cells above total nuclei. ** $p < 0.01$, *** $p < 0.001$ versus control.

Supplementary Table 1: Primer sequences used to measure mRNA expression.

**MHC1**	F CTC AAG CTG CTC AGC AAT CTA TTT<br>R GGA GCG CAA GTT TGT CAT AAG T
**MHC2A**	F AAG CGA AGA GTA AGG CTG TC<br>R GTG ATT GCT TGC AAA GGA AC
**MHC2B**	F CAC CTG GAG CGG ATG AAG AAG AAC<br>R GTC CTG CAG CCT CAG CAC GTT
**GPR56**	F CTG CGG CAG ATG GTC TAC TTC<br>R CCA CAC AAA GAT GTG AGG CTC
**COL3A1**	F GCA CAG CAG TCC AAC GTA GA<br>R TCT CCA AAT GGG ATC TCT GG
**PGC-1a total**	F TGA TGT GAA TGA CTT GGA TAC AGA CA<br>R GCT CAT TGT TGT ACT GGT TGG ATA TG
**PGC-1a1**	F GGA CAT GTG CAG CCA AGA CTC<br>R CAC TTC AAT CCA CCC AGA AAG CT
**PGC-1a4**	F TCA CAC CAA ACC CAC AGA AA<br>R CTG GAA GAT ATG GCA CAT
**IGF1**	F GGC ATT GTG GAT GAG TGT TG<br>R TCT CCT TTG CAG CTT CGT TT
**IGF2**	F CGC TTC AGT TTG TCT GTT CG<br>R AGG TAG ACA CGT CCC TCT CG
**18S**	F GTA ACC CGT TGA ACC CCA TT<br>R CCA TCC AAT CGG TAG TAG CG

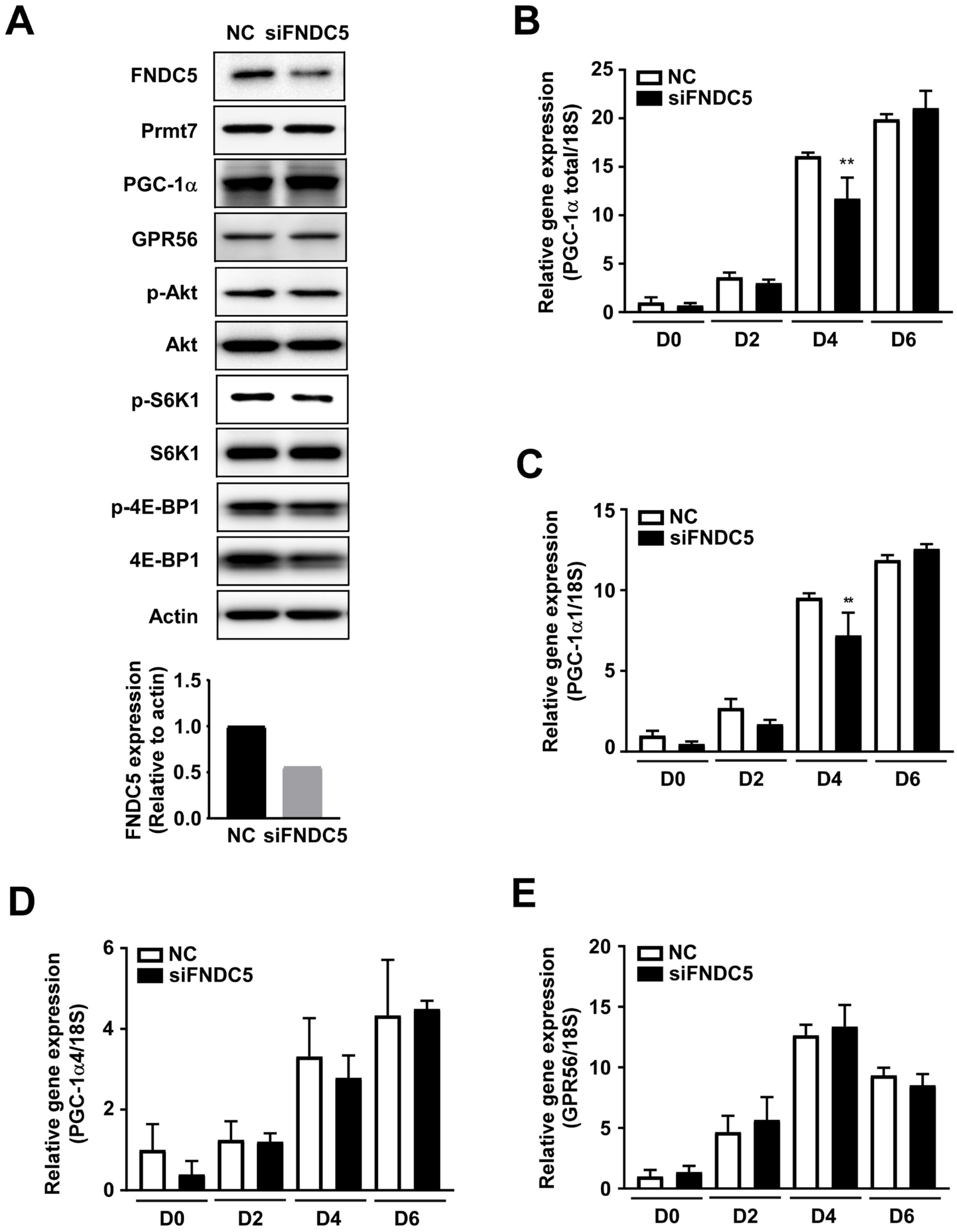

Supplementary Figure 5: Effect of FNDC5 knockdown on PGC-1α and GPR56 expression. (**A**) FNDC5 did not change protein expression levels of Prmt7, PGC-1α, GPR56, or phospho-Akt. Phospho-S6K1 and 4E-BP1 were attenuated by FNDC5 knockdown. C2C12 cells were transfected with siRNA against FNDC5 for 48 hours. FNDC5 protein expression was quantified by Image J. (**B-E**) FNDC5 knockdown did not alter the mRNA expression of total PGC-1α, PGC-1α1, PGC-1α4 and GPR56. C2C12 cells were transfected with siFNDC5 or NC and differentiated for 2, 4, or 6 days. mRNA expression was quantified by qPCR. Data are expressed as means ± S.D. ** $p < 0.01$ versus NC.
